# The Interplay between Oncogenic Signaling Networks and Mitochondrial Dynamics

**DOI:** 10.3390/antiox6020033

**Published:** 2017-05-17

**Authors:** Sarbajeet Nagdas, David F. Kashatus

**Affiliations:** Department of Microbiology, Immunology and Cancer Biology, University of Virginia Health System, Charlottesville, VA 22908, USA; sn9mn@virginia.edu

**Keywords:** mitochondrial dynamics, oncogenic signaling, cancer

## Abstract

Mitochondria are dynamic organelles that alter their organization in response to a variety of cellular cues. Mitochondria are central in many biologic processes, such as cellular bioenergetics and apoptosis, and mitochondrial network morphology can contribute to those physiologic processes. Some of the biologic processes that are in part governed by mitochondria are also commonly deregulated in cancers. Furthermore, patient tumor samples from a variety of cancers have revealed that mitochondrial dynamics machinery may be deregulated in tumors. In this review, we will discuss how commonly mutated oncogenes and their downstream effector pathways regulate the mitochondrial dynamics machinery to promote changes in mitochondrial morphology as well as the physiologic consequences of altered mitochondrial morphology for tumorigenic growth.

## 1. Introduction

Mitochondria are double-membrane-bound organelles that are central to a variety of cellular physiological processes, such as the regulation of bioenergetics, the maintenance of cellular oxidation-reduction (redox) status, and the execution of apoptosis. Structurally, mitochondria form networks, and consist of an outer mitochondrial membrane (OMM), intermembrane space, inner mitochondrial membrane (IMM), and matrix. The mitochondrial network can exist along a spectrum of morphologies from a highly interconnected, elongated network to a highly fragmented, punctate morphology. Under homeostatic conditions, the mitochondrial network constantly undergoes mitochondrial fusion and fission events, and the relative balance of these activities results in a mixture of interconnected, intermediate, or fragmented morphology. Different cellular cues and signals converge upon the regulators of mitochondrial dynamics to alter mitochondrial morphology, and the morphological state confers properties appropriate for the conditions. For example, elongated mitochondrial morphology allows for complementation of damaged mitochondrial components under conditions of stress, while mitochondrial fragmentation allows for easier transport of mitochondria along the cytoskeleton to areas of high energy demand [1,2]. Over the years, numerous studies have shown that disruptions in normal mitochondrial dynamics are associated with a host of human pathologies, such as neurodegenerative disorders and cardiomyopathies [3]. Similarly, a growing number of studies have demonstrated the links between abnormal mitochondrial dynamics and various types of cancers. Here, we will review recent studies that demonstrate how common oncogenic signaling pathways converge upon mitochondrial dynamics regulators and contribute to the tumorigenic phenotypes. 

## 2. Mitochondrial Dynamics Machinery

**Figure 1:**
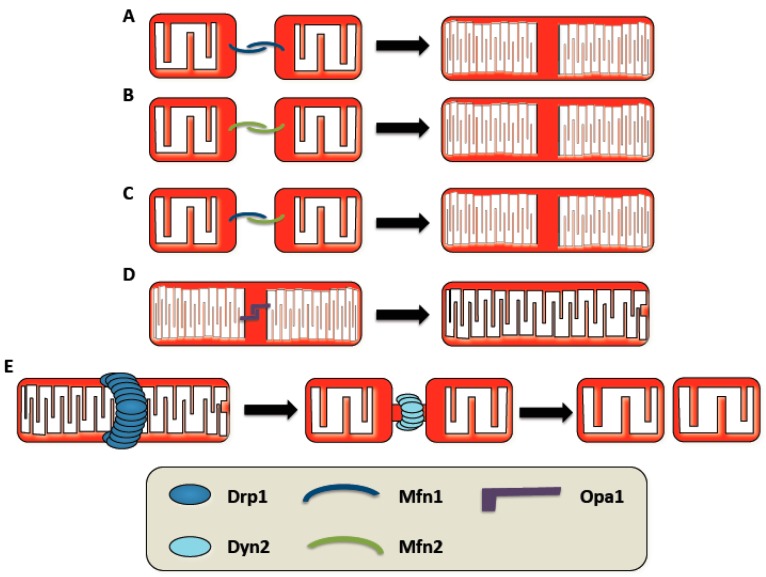
The primary mitochondrial dynamics machinery responsible for mitochondrial membrane fusion and fission. Outer mitochondrial membrane fusion is primarily mediated by mitofusin 1 and 2 (Mfn1 and Mfn2). (**A**–**C**) Homotypic Mfn1 and Mfn2 complexes or heterotypic Mfn1:Mfn2 complexes execute outer membrane fusion. (**D**) Optic atrophy 1 (Opa1) is the large guanosine triphosphate hydrolase (GTPase) that mediates inner mitochondrial membrane fusion. Inner mitochondrial membrane fusion is typically coupled with outer mitochondrial membrane fusion. (**E**) Dynamin-related protein 1 (Drp1) oligomerizes as spirals around the mitochondrial membrane. Upon GTP hydrolysis, the mitochondria are greatly constricted which serves as the platform for Dynamin-2 (Dyn2) to complete fission of the mitochondrial unit.

Mitochondrial fusion and fission are highly conserved processes that are primarily mediated by large GTPases. Mitofusin 1 (Mfn1) and mitofusin 2 (Mfn2) execute OMM fusion in mammals, while Optic atrophy 1 (Opa1) performs IMM fusion. Conversely, Dynamin-related protein 1 (Drp1), along with Dynamin-2, mediate mitochondrial fission for both the inner and outer membranes (Figure 1). Here, we will provide a brief overview of these mitochondrial dynamics processes as they have been reviewed recently [1,4].

### 2.1. Mitochondrial Outer and Inner Membrane Fusion

Two mitochondrial outer membranes that are in close proximity to one another require mitofusins on both opposing membranes in order for OMM fusion to occur [5]. Mitofusin 1 and 2 can form either homotypic or heterotypic complexes with each other to tether the membranes; interestingly, heterotypic complexes are more efficient at OMM fusion than homotypic complexes [6,7,8]. Structural studies suggest that the physical fusion event occurs by pulling together opposing membranes analogous to soluble N-ethylmaleimide-sensitive factor attachment protein receptor (SNARE) proteins [7]. A recent study using crystal structures of engineered Mfn1 showed that GTPase domain dimerization during GTP hydrolysis is needed for OMM fusion [9]. The extensive mitochondrial fragmentation seen in either Mfn1^−/−^ or Mfn2^−/−^ cells can be rescued by the overexpression of Mfn2 or Mfn1, respectively [10,11]. Despite this, it should be noted that Mfn1 and Mfn2 are not completely functionally redundant. In addition to its role in mitochondrial fusion, Mfn2 also tethers mitochondria to the endoplasmic reticulum (ER) and helps regulate mitochondrial calcium levels originating from the ER [12].

The mitofusins are regulated by a variety of posttranslational modifications as well as through direct associations with other proteins. In combination, these regulatory mechanisms dictate which mitochondrial contacts will result in mitochondrial fusion [13,14]. Mfn1 and Mfn2 activity can be modified by specific phosphorylation events and ubiquitination of both proteins can result in degradation [15]. One of the best-characterized examples of this regulation is following the recruitment of Parkin to depolarized mitochondria. Parkin directly ubiquitinates Mfn2, leading to its degradation and preventing the fusion of the damaged mitochondrion to a healthy one [16,17]. Mfn2 can also be regulated through acetylation, and its deacetylation results in its activation in response to nutrient deprivation [18]. Eura et al. identified and characterized Mitofusin binding protein (MIB) as a novel binding partner of Mfn1 and a negative regulator of Mfn1 activity [19]. Additionally, it has been demonstrated that in healthy cells, the pro-apoptotic Bcl-2 family member, Bax, interacts with Mfn2 and stimulates mitochondrial fusion [20,21]. 

Optic atrophy 1 (Opa1) is the large GTPase that mediates IMM fusion sequentially following OMM fusion [6]. There are numerous splice variants of Opa1 and it can be further processed through specific proteolytic cleavage events [22]. Cleavage of Opa1 represents a major mechanism of its regulation. The long isoform of Opa1 has been shown to be necessary for IMM fusion, although the exact mechanism of IMM fusion remains unclear [23]. Processing of Opa1 by the OMA1 peptidase or the *i*-AAA protease YME1L not only inhibits its inner membrane fusion activity, but can also directly promote mitochondrial fragmentation [24,25]. Opa1 has also been shown to mediate cristae remodeling, which is central in maintaining proper oxidative phosphorylation and apoptosis, independent of its IMM fusion function [26,27]. 

Mediating mitochondrial fusion has significant consequences for the maintenance of proper health and development, as evidenced by the identification of Mfn1/2 or Opa1 dysfunction in numerous pathologies. Mfn2 mutations have been identified in patients with Charcot-Marie-Tooth Disease Type 2A (CMT2A), a neuromuscular disorder that impacts motor nerve conduction [28,29]. Mfn2 deficiency has also been associated with human models of pulmonary arterial hypertension [3,30]. To date, there have been no reported pathologies caused by or associated with mutations or dysfunction in Mfn1 [3]. Genetic deletion of Mfn1 and Mfn2 are both embryonic lethal in mice, indicating the critical role of mitochondrial fusion during development [10]. Interestingly, Mfn2^−/−^ mice exhibit embryonic lethality due to improper placental development while the cause of Mfn1^−/−^ mice’s embryonic lethality remains poorly understood. This divergence in pathologies illustrates that Mfn1 and Mfn2 are functionally distinct GTPases. Mutations in the OPA1 gene are the most common cause of dominant optic atrophy, an optic neuropathy, while polymorphisms in OPA1 are associated with hypertension [3,31,32]. Additionally, Opa1 is critical for proper mammalian development as homozygous deletion of Opa1 is embryonic lethal, although the exact cause of this lethality remains unknown [33]. 

### 2.2. Mitochondrial Fission

While mitochondrial fusion utilizes separate GTPases to fuse the OMM and IMM, it is thought that both IMM and OMM fission are mediated by the GTPase Drp1 [34]. While the central role of Drp1 in mitochondrial division has been appreciated for many years, recent studies have highlighted the complexity and coordination of its regulation. Prior to Drp1 recruitment to the mitochondrial outer membrane, constriction of the membrane occurs at specific sites marked by contact between the OMM and the endoplasmic reticulum (ER) [35]. Dimers and tetramers of Drp1 are then recruited from the cytosol to a set of specific adaptors associated with the OMM [36]. At the mitochondria, Drp1 oligomerizes into higher-order spirals, which triggers GTP hydrolysis and subsequent constriction of the mitochondria [4,37]. Drp1-driven constriction is sufficient to narrow the mitochondrial diameter but insufficient to promote its complete severing [37,38]. Recently, Lee et al. demonstrated that dynamin-2 is recruited to sites of Drp1-mediated constriction and severs the mitochondria to complete mitochondrial fission [39]. 

Drp1 activity is regulated by a variety of post-translational modifications that can either activate or inhibit its ability to promote mitochondrial fission. A key modification that promotes Drp1 activity and mitochondrial fission is phosphorylation at S616, which can be mediated by a number of different kinases, including protein kinase C δ (PKCδ) [40]. Conversely, the inhibition of Drp1 activity under a variety of conditions is mediated through phosphorylation at residue S637, which can be targeted by protein kinase A (PKA) [41,42]. Calcineurin is a Drp1 phosphatase that removes the inhibitory S637 phosphorylation, leading to increased fission activity [42,43]. In neuronal systems, Drp1 has been shown to be *S*-nitrosylated although this post-translational modification does not impact Drp1 activity directly [14,44]. Drp1 can additionally be regulated by sumoylation and ubiquitination, which contribute to Drp1 stability [45,46]. 

Like the fusion machinery, proper Drp1 function is essential for proper development and physiology. Underscoring this point, a patient with a germ-line, dominant negative Drp1 mutation suddenly died 37 days after birth, failing to thrive and exhibiting various neurologic and metabolic dysfunctions [3,47]. Additionally, the whole-animal knockout of Drp1 leads to embryonic lethality in mice at E11.5 due to the absence of the trophoblast giant cell layer of placental development and dysfunctional cardiomyocytes. This phenotype suggests Drp1 plays an important role in ensuring proper oxygen and nutrient exchange during mammalian development [48]. Interestingly, Mfn2^−/−^ mice also lack the trophoblast giant cell layer, indicating that proper mitochondrial dynamics may be critical in proper placental, and consequently, cardiovascular development. Paralleling what has been observed in the Drp1-mutant patient, Wakabayashi et al. demonstrated that Drp1 is necessary for cerebellar development. Further underscoring the importance of Drp1 in organismal health, Drp1 has been implicated in different cardiovascular, neurologic, and metabolic diseases [3,49,50]. 

Given the importance of mitochondrial dynamics in normal development and various pathologies, it is unsurprising that the machinery is also implicated in a variety of cancers [51,52,53]. In this review, we will discuss how common oncogenic signaling pathways regulate mitochondrial fusion and fission and how this regulation may impact cancer cell physiology. 

## 3. MAPK Signaling

The Ras family of small GTPases utilizes numerous downstream effector pathways to mediate its diverse biological functions, such as to promote proliferation and suppress apoptosis [54]. Ras is mutated in up to a third of all cancers, causing it to be locked in its active conformation and to activate a host of downstream effector pathways [55]. One of best-understood Ras effector pathways is the mitogen activated protein kinases (MAPK) pathway. In addition to mutations in Ras, the MAPK pathway can also become activated due to mutations in the Ras-binding kinase rapidly accelerated fibrosarcoma (Raf) or to inactivating mutations in negative regulators of MAPK such as neurofibromin 1 (NF1) [56]. In wildtype cells, Ras activity leads to engagement and activation of Raf, which in turn activates mitogen activated protein kinase kinase (MEK), which activates extracellular signal regulated kinase (Erk). Active Erk is able to phosphorylate a variety of targets, including other kinases and transcription factors that ultimately contribute to the diverse physiological functions of Ras.

The MAPK pathway regulates many biological processes that are linked to mitochondrial function and whose dysregulation are hallmarks of cancer, such as evading apoptosis and altering cellular metabolism, suggesting that this pathway may directly impact mitochondrial function [54,57,58,59]. Consistent with this, Erk1 was demonstrated to phosphorylate Drp1 to promote mitochondrial fission in an in vitro kinase assay [60]. More recently, our lab, along with the lab of Jerry Chipuk, showed that Drp1 is directly activated via Erk2-mediated S616 phosphorylation and that mitochondria are fragmented downstream of the MAPK pathway in two independent Ras-MAPK-driven cancer systems [61,62]. Furthermore, constitutively active oncogenic mutants Ras^G12V^ and B-Raf^V600E^ both cause an increase in Drp1 mRNA levels, which can be reversed upon pharmacologic inhibition of the mutant v-Raf murine sarcoma viral oncogene homolog B (B-Raf), MEK, or ERK [62]. Using xenografts of immortalized human embryonic kidney (HEK) cells with activated Ras, it was demonstrated that S616 phosphorylation and activation of Drp1 are required for Ras-induced tumor growth [61]. Finally, Drp1 is phosphorylated in tumor samples from both MAPK-driven melanoma patients and pancreatic ductal adenocarcinoma patients [61,62]. 

In addition to regulating mitochondrial fission, the MAPK pathway has also been shown to regulate mitochondrial fusion. Using mouse embryonic fibroblasts (MEFs), Pyakurel et al. showed that Erk phosphorylates Mfn1 at T562, which inhibits Mfn1 activity and results in more fragmented mitochondria [63]. Both genetic approaches and epidermal growth factor stimulation to activate MEK lead to this inhibitory phosphorylation. While it is clear from these studies that MAPK signaling can regulate both the fusion and fission machinery, there is also evidence that mitofusins regulate Ras-MAPK signaling. In rat vascular smooth muscle cells, Mfn2 was shown to bind and sequester Ras, resulting in MAPK inhibition [64]. Additionally, studies performed in B cell lymphoma cell lines indicated that Mfn1 can interact with Ras [65]. Surprisingly, introduction of either wild-type Mfn2 or an Mfn2 mutant incapable of binding to Ras into Mfn2^−/−^ MEFs was able to revert the fragmented mitochondrial morphology seen in Mfn2^−/−^ MEFs to a more intermediate mitochondrial morphology [12]. Furthermore, the introduction of constitutively active Ras or MEK into wild-type MEFs did not alter mitochondrial morphology in this system [66]. These results suggest that the Mfn2-Ras signaling axis and its impact on mitochondrial morphology may be context dependent.

In addition to these roles in mitochondrial fission and OMM fusion, the MAPK pathway may also regulate IMM fusion through Opa1. For example, the treatment of human hepatocellular carcinoma cell lines and xenografts with sorafenib, a Raf inhibitor, led to decreased expression of Opa1, but these results should be interpreted with caution as sorafenib has been shown to inhibit the activity of multiple different kinases [67].

This relationship between the Ras-MAPK pathway and the mitochondrial dynamics machinery raises the question of how this regulation contributes to cancer. A potential oncogenic role of MAPK-regulated mitochondrial dynamics is in the initial tumor-establishing stages of tumorigenesis. In pancreatic cancer, evidence suggests that a malignant tumor arises in a step-wise fashion from pre-neoplastic lesions [68]. A recent study demonstrated that V-Ki-ras2 Kirsten rat sarcoma viral oncogene homolog (KRas) induced mitochondrial reactive oxygen species (ROS) promote the formation of pancreatic pre-neoplastic lesions [69]. It is tempting to speculate that one role of KRas- or MAPK-induced mitochondrial fission is to promote mitochondrial ROS generation that can subsequently promote tumorigenesis.

Another possible role for MAPK regulation of Drp1 may be to initiate changes in tumor cell differentiation. Prieto et al. demonstrated that Erk activation of Drp1 and subsequent mitochondrial fragmentation is necessary during the early stages of cellular reprogramming to promote the formation of induced pluripotent stem cells [70]. Cellular reprogramming and dedifferentiation occur in numerous cancers contributing to a tumor’s intra- and inter-tumoral heterogeneity and are associated with a worse pathologic grade [71,72]. 

Although MAPK regulation of Drp1 promotes processes that favor tumorigenesis, Mfn2 regulation of MAPK appears to inhibit tumorigenesis, at least in the systems that were tested. Mfn2 was shown to have antiproliferative effects through knockdown and rescue studies in B cell lymphoma cell lines [65]. These effects were mediated through Mfn2 interaction with Ras and Raf and subsequent inhibition of the MAPK pathway. In normal physiology, this sequestration of Ras may provide a mitochondrial brake on Ras-induced mitogenic signaling in order to ensure proper cell cycle progression. However, given the role of mitogenic signaling in tumorigenesis, this sequestration would be disadvantageous for cancers. Indeed in gastric cancers, a cancer that can harbor Ras mutations, there is decreased Mfn2 expression, suggesting possible selective pressure against the sequestration and inhibition of a mitogenic signal [73,74]. 

## 4. PI3K-Akt Signaling

Another well-studied Ras effector pathway is the phosphoinositide 3-kinase (PI3K)/protein kinase B (Akt) signaling axis. Active Ras binds to the PI3K catalytic subunit alpha (PI3KCA), which results in PI3K activation. Activated PI3K generates phosphatidylinositol-3,4,5-triphosphate (PIP3), which can activate a host of downstream kinases including Akt [56,75]. In addition to Ras mutation, this signaling axis can also be hyperactivated by improper regulation of PI3K pathways regulators, such as inactivation of the PI3K inhibitor phosphatase and tensin homolog (PTEN) or aberrant activation of PI3KCA [75,76]. Mutations, copy number alterations, and epigenetic regulation of PTEN and PI3KCA are commonly found in several different malignancies [75,76]. Studies have indicated that Akt can regulate numerous biologic processes, such as cell proliferation and growth, central to tumorigenesis [75].

For our discussion, we will briefly review some of major signaling components implicated in those biologic processes downstream of Akt. Akt signaling can promote cellular proliferation through antagonizing the negative regulators of cyclins and cyclin-dependent kinases such as p21, the cyclin-dependent kinase inhibitor of CDK1, and p27, an inhibitor of CDK2 and CDK4 [75,77]. The emerging primary regulator of cellular growth downstream of Akt signaling is the mammalian target of rapamycin (mTOR). Much of mTOR’s signaling occurs while complexed with other proteins, primarily mTORC1 and mTORC2 [78]. mTORC1 can promote its effects on cellular growth by propagating signals that activate transcriptional programs for organelle biogenesis and macromolecular synthesis [78]. Interest in mTORC2 is rapidly rising and it is suggested to function as a positive regulator and full activator of Akt, which can regulate cell cycle progression, survival, and metabolism [79].

A handful of recent studies indicate that the PI3K/Akt/mTOR pathway regulates mitochondrial dynamics. Tondera et al. demonstrated that PI3K promotes the expression of mitochondrial protein, 18kDa (MTP18), and that MTP18 promotes mitochondrial fragmentation [80]. In addition, using an Alzheimer’s disease system, Kim et al. demonstrated that Akt directly phosphorylates and activates Drp1, which results in Drp1-mediated mitochondrial fission. They showed that Ca^2+^/calmodulin-dependent protein kinase II (CAMKII) promotes Akt activation, which ultimately activates Drp1 [81]. Conversely, Xie et al. showed that CAMKII phosphorylates Drp1 directly on its inhibitory S637 residue [82]. These opposing CAMKII-mediated signals on Drp1 suggest that in PI3K-driven cancer cells, Drp1 phosphorylation status is skewed more towards promoting fission. In addition, PTEN, which antagonizes PI3K signaling, was shown to support canonical Wnt-induced mitochondrial fusion, consistent with a pro-fission role for PI3K/Akt. [83]. In contrast to the data indicating that PI3K/Akt activity promotes mitochondrial fragmentation, Caino et al. showed that reactivation of Akt and mTOR following PI3K inhibition led to increased mitochondrial fusion [84]. However, they showed that knockdown of Akt or mTOR in the absence of pharmacological inhibition of PI3K did not impact mitochondrial morphology in their prostate cancer cell lines [84]. This suggests that there may be additional factors that influence PI3K/Akt regulation of mitochondrial morphology, especially in the context of drug treatment. 

Similar to the MAPK pathway, Akt signaling is also subject to regulation by Mfn2. Using rat vascular smooth muscle cells, Guo et al. demonstrated that Mfn2 inhibits Akt activation earlier and to a greater extent than it does Erk1/2 [85]. They also demonstrated that Mfn2 inhibition of Akt and not its impact on mitochondrial fusion, is necessary for Mfn2 to induce apoptosis in vascular smooth muscle cells. Another study showed that Mfn2 can inhibit Akt signaling through inhibition of the mTORC2 complex, which results in decreased colony formation and xenograft growth [86]. This study found that Mfn2 binds to rapamycin-insensitive companion of mTOR (RICTOR), a member of the mTORC2 complex, and is required for the inhibition of mTORC2 signaling and Akt activation. These findings are similar to Chen et al.’s findings with Ras in lymphoma cells, showing that direct binding of Mfn2 to these signaling proteins promote anti-proliferative effects. Like MAPK, Mfn2 antagonism of Akt would be disadvantageous for cancer cell survival. Consistent with this, Xu et al. showed that patients with low Mfn2 levels had poorer prognoses, indicating more aggressive tumors, and they demonstrated that Mfn2 loss enhanced tumor growth in breast and lung cancer xenograft systems [86].

## 5. RalGEF Signaling

In addition to the previously described Ras effector pathways, the Ras-like protein (Ral)/Ras-like guanine nucleotide exchange factor (RalGEF) signaling network has been shown to regulate mitochondrial dynamics [87]. Activated Ras binds to RalGEFs, which promote the activation of the Ral GTPases RalA and RalB. Increased Ral activity has been reported in human pancreatic, bladder, and colon cancer cell lines and tissues [88,89,90,91] and RalA is required for the tumorigenic growth of many Ras-driven cancer cell lines [88,92]. Like MAPK and PI3K, RalA can also drive changes in mitochondrial morphology as RalA promotes activation and mitochondrial recruitment of Drp1 during mitosis [93]. These data suggest that one potential pro-tumorigenic role for RalA is in the induction or maintenance of a fragmented mitochondrial phenotype. 

## 6. Biologic Effects of Ras Effector Pathway-Mediated Mitochondrial Fission

**Figure 2:**
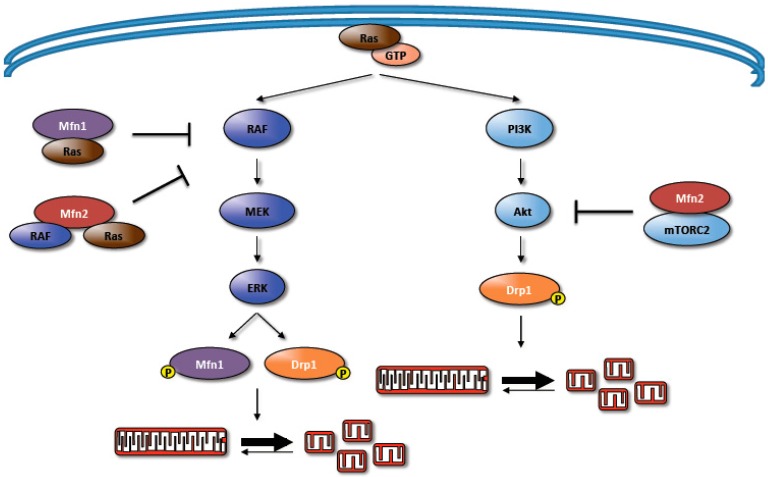
The reciprocal regulation of the mitochondrial dynamics machinery by the mitogen activated protein kinase (MAPK) and phosphoinositide 3-kinase (PI3K)/protein kinase B (Akt) signaling pathways. The activated MAPK pathway promotes mitochondrial fission by activating Drp1 as well as inhibiting Mfn1 activity. Conversely, Mfn1 has been shown to interact with Ras while Mfn2 has been shown to interact with either Ras or Raf; these interactions have been shown to inhibit MAPK activity and some of its physiological consequences. Activated Akt can directly phosphorylate and activate Drp1 and promote mitochondrial fission. Additionally, Mfn2 can interact with the mammalian target of rapamycin complex 2 (mTORC2) complex, via interactions with rapamycin-insensitive companion of mTOR (RICTOR), which inhibits PI3K/Akt activity.

Collectively, the data suggest that activated Ras and the subsequent activation of Ras effector pathways cause cancer cells to acquire and maintain fragmented mitochondrial morphology (Figure 2). This suggests that fragmented mitochondria, at least in the context of Ras mutation, confer some set of advantageous biologic processes that promote tumor growth. Several studies have uncovered roles for mitochondrial fusion and fission in numerous biologic processes that could potentially contribute to tumor growth. Here, we will provide a brief overview of the role of fragmented mitochondria in cell proliferation, apoptosis, cellular bioenergetics, and cell motility and migration.

### 6.1. Cell Proliferation

Progression through the cell cycle and the coordination of mitochondrial morphology are tightly linked, with disruptions in either process affecting the other. Mitochondrial fission is critical for proper segregation of mitochondria to daughter cells [94]. Additionally, the loss of Drp1 is found to slow the growth of MEFs and promote decreased levels of Ki67, a marker of cell proliferation, in vivo [48]. The cyclin dependent kinase Cdk1, which is activated during the S phase and promotes progression through to the M phase, directly activates Drp1 via phosphorylation of its S616 residue. At the G1-S transition point, mitochondria become hyperfused, potentially through reduced Drp1 activity [95]. Prolonged mitochondrial fusion is detrimental to the cell since mitotic chromosomal alignment defects arise [95]. Qian et al. found that inhibition of Drp1 results in cell cycle arrest at the G2/M transition due to replication stress [96]. They also demonstrated that forced mitochondrial fusion induces inappropriate cyclin E expression, triggering activation of the G2/M checkpoint [96]. Finally, they showed that loss of Drp1 induces chromosome instability and centrosome amplification. Collectively, these data indicate that aberrant mitochondrial fusion during the cell cycle can result in delayed cell division without high fidelity genome replication. From the perspective of a cancer cell, these data suggest that inhibition of mitochondrial fragmentation or promotion of mitochondrial fusion may slow down cellular proliferation and increase the likelihood of genetic instability. Consistent with this hypothesis, Rehman et al. demonstrated that either the inhibition of Drp1 or expression of Mfn2 impairs a lung cancer cell’s ability to progress through the cell cycle and decreases tumor growth in a xenograft model [51].

### 6.2. Apoptosis

The interplay between mitochondrial dynamics, the dynamics machinery, and apoptosis are numerous and excellently summarized in other reviews [57,97,98]. Here, we will briefly review how mitochondrial dynamics appears to contribute to the intrinsic (mitochondrial) apoptotic pathway. In this pathway, an apoptotic stress induces cleavage of Bid to form truncated Bid (tBid), which can interact with B-cell lymphoma 2 (Bcl-2) family proteins to promote OMM permeablization. tBid can either activate the proapoptotic proteins Bcl-2 family members Bax and Bak or can inactivate anti-apoptotic Bcl-2 family members, which ultimately results in Bax and Bak activation. Bax and Bak can both permeablize the OMM and cause cristae disorganization, causing cytochrome C release to the cytosol. Cytosolic cytochrome C interacts with Apaf-1, which triggers the activation of the caspase cascade and ultimately the execution of apoptosis. 

Almost invariably, the execution of apoptosis corresponds with fragmentation of the mitochondria. Correspondingly, Bax and Bak were found to colocalize with Drp1 during the initial stages of apoptosis at future sites of fission [20,99]. In addition, shRNA mediated knockdown of Drp1 has been shown to delay cytochrome C release in HeLa cells, suggesting that Drp1 is needed for the progression of apoptosis [100]. However, Drp1^−/−^ MEFs and embryonic stem cells are able to undergo apoptosis, suggesting that Drp1 is not required for apoptosis execution [48]. Despite the apparent dispensability of Drp1 in apoptosis, Drp1^−/−^ MEFs exhibit apparent mitochondrial fragmentation after cytochrome C release [94]. This suggests that mitochondrial fission, regardless of Drp1 status, may be needed for apoptosis progression at a stage after OMM permeabilization. 

The mitochondrial fusion components are also intimately intertwined with the apoptotic machinery. Karbowski et al. demonstrated that mitochondrial fusion is blocked during apoptosis, suggesting an anti-apoptotic role for mitochondrial fusion [99]. Consistent with this, the overexpression of Mfn1/2 inhibits apoptosis while cells with knockdown of Mfn1/2 were more sensitive to apoptosis [101]. Furthermore, the expression of a mutant Mfn2 that promotes enhanced mitochondrial fusion relative to wild-type Mfn2 results in decreased apoptosis relative to the Mfn2 wild-type [102]. In addition, the downregulation of Opa1 induces mitochondrial fragmentation and apoptosis independently of Mfn1/2 without an apoptotic stimulus [26,103]. Collectively, these studies suggest that mitochondrial fusion antagonizes apoptosis.

There is also evidence that the apoptotic machinery can influence mitochondrial dynamics. For example, Bax and Bak are required for mitochondrial fusion in healthy cells with no apoptotic stimulus [21]. Karbowski et al. also demonstrated that Mfn2 is localized to and mobile along the OMM in Bax/Bak double knockout MEFs and that ectopic expression of Bax in those MEFs redistributed Mfn2 into foci, similar to wild-type MEFs, and reduced Mfn2 mobility in the membrane [21]. This shows that Bax regulates Mfn2 mobilization and focus formation, which occur at sites of mitochondrial fusion [21]. It has also been shown that soluble Bax can promote mitochondrial fusion through interactions with homotypic Mfn2 complexes [8]. Hoppins et al.’s findings also suggest that Mfn2 can sequester soluble Bax, rendering cells less prone to apoptosis. Regardless, collectively these studies suggest that mitochondrial fragmentation is permissive for apoptosis under stressed conditions and that the apoptotic machinery promotes mitochondrial fusion to prevent apoptosis for a healthy cell.

Whether the increased fragmentation observed in Ras-driven tumors affects apoptotic signaling in those tumors, and whether the potential effects on apoptosis are pro- or anti-tumorigenic, remains unclear. Most of the literature would support a model in which the fragmentation is pro-apoptotic and perhaps represents an attractive therapeutic vulnerability that distinguishes tumor cells from normal cells. However, Renault et al. demonstrated that excessive mitochondrial fragmentation fails to support Bax-mediated OMM permeabilization, suggesting that the tumors with highly fragmented mitochondria might be protected [104]. Furthermore, although mitochondrial fragmentation is associated with apoptosis, many of the aforementioned studies demonstrate that Drp1-mediated mitochondrial fragmentation is not sufficient for apoptosis execution. Clearly, more thorough investigation into the relationship between mitochondrial dynamics and apoptotic sensitivity in tumor cells is needed.

### 6.3. Metabolism

Given the critical role of mitochondria in oxidative metabolism, it is not surprising that mitochondrial dynamics and morphology can influence many aspects of cellular metabolism. It is well established that cancers, including Ras-driven cancers, alter cellular bioenergetics that promote tumorigenesis [105,106]. There have been many excellent reviews that have carefully examined the regulatory role of mitochondrial dynamics on cellular bioenergetics [58,107,108,109,110]. In light of this, we will limit the scope of this discussion to connections between mitochondrial dynamics and aerobic glycolysis as well as autophagy, two arms of cellular bioenergetics particularly prevalent in Ras driven cancers.

The most well-documented metabolic alteration in cancer cells is the Warburg effect, which is the preferential utilization of glycolysis over oxidative phosphorylation under aerobic conditions [105,106]. There is growing experimental support for the notion that mitochondrial fusion promotes more efficient oxidative phosphorylation and thus increased adenosine triphosphate (ATP) production. Studies in mammals and lower organisms indicate that the loss of mitochondrial fusion, even a partial loss, results in a stochastic loss of membrane potential, and consequent loss of mitochondrial functionality [10,58]. In cells lacking both Mfn1 and Mfn2, there is compromised mitochondrial respiration [111]. Furthermore, oxidative phosphorylation can regulate IMM fusion independent of OMM fusion, which, it was suggested, may be a mechanism to ensure that energy demands are met [27]. More recent studies support this notion. For example, Mitra et al. demonstrated that the hyperfused mitochondria observed during the G1-S phase produce more adenosine triphosphate ATP than mitochondria in cells at other phases in the cell cycle [95]. Collectively, the current data support a model in which fragmented mitochondria, like those observed in cancers driven by Ras and its effector pathways, facilitate a more glycolytic metabolism. 

Another method cancer cells use to acquire energy and macromolecules is autophagy. In addition, autophagy can remove dysfunctional cellular components, including mitochondria, to maintain overall cellular health [112,113]. Autophagy can be induced by Ras and is required for Ras-driven tumorigenesis [114]. Additionally, mitophagy is able to provide energy during the early stages of Ras-mediated transformation [115]. Studies have shown that mitochondrial elongation, through the inhibition of Drp1 or overexpression of Opa1 can reduce mitophagy levels [13]. The inhibition of mitophagy through mitochondrial elongation has also been observed under conditions of starvation [116]. Interestingly, AMPK, a known inducer of autophagy, is required for cells to undergo mitochondrial fragmentation in response to mitochondrial damage that reduces cellular ATP levels [117,118]. AMPK-mediated mitochondrial fragmentation may serve to initiate mitophagy to sequester damaged portions of the mitochondrial network. Collectively, these studies suggest that mitochondrial fragmentation observed in Ras-driven cancers would be permissive for increased mitophagy, which would support tumor growth. 

### 6.4. Cell Motility and Migration

Mitochondria in mammalian cells make contacts with, and are functionally regulated by, both the actin and microtubule cytoskeletons. De Vos et al. demonstrated that actin facilitates Drp1 recruitment to the mitochondria to promote mitochondrial fission [119] and it was also shown that actin filaments accumulate at future sites of fission prior to Drp1 and increase the rate of fission [120]. Korobova et al. demonstrated that actin filaments localized at the sites of ER-mitochondria constriction sites are required for efficient mitochondrial fission [121]. These links between actin dynamics and Drp1 prompted the investigation of whether Drp1 and mitochondrial fission play a role in cell motility and migration, biological processes with high energy demands that are dependent on actin dynamics. The inhibition of mitochondrial fragmentation, either by inhibiting Drp1 via RNAi or overexpressing Mfn1, leads to decreased migration, invasion, and lamellipod formation in breast cancer cell lines [122]. Similarly, pharmacological or genetic inhibition of Drp1 in thyroid cancer cell lines decreases cell migration and invasion [123]. These results were further recapitulated in glioblastoma and glioma cell lines [124,125]. Yin et al. showed that knockdown of Drp1 reduces levels of Ras homolog family member A (RhoA) and Rho-associated, coiled-coil containing protein kinase 1 (ROCK1), critical regulators of actin cytoskeletal dynamics and cell motility [125]. Interestingly, ROCK1 was found to phosphorylate and activate Drp1, triggering mitochondrial fission under hyperglycemic conditions in mouse podocytes and endothelial cells [126]. Although this study did not explore cell motility and migration, this finding suggests that Drp1 and Drp1-mediated mitochondrial fission may promote a feed-forward regulatory cycle with ROCK1 to promote cell motility and migration. 

## 7. MYC Signaling

The transcription factor Myc is activated downstream of many different signaling pathways, including PI3K/Akt and MAPK, and it regulates a number of physiological processes important for cancer [59,127]. ChIP-seq studies have identified numerous Myc-responsive genes implicated in ribosomal biogenesis, nucleotide metabolism, and DNA replication, processes that are important for cell division [128]. Myc also regulates metabolic genes that drive the glycolytic shift characteristic of the Warburg effect [128,129] and it can alter cellular sensitivity to various apoptotic stimuli, depending on the cellular context [130]. Though infrequently mutated in cancer, Myc is overexpressed in upwards of 50% of all cancers due to chromosomal translocation (e.g., Burkitt’s lymphoma), gene amplification, or aberrant cellular signaling regulation [131,132]. Furthermore, increased expression of Myc correlates with poor patient survival [133]. Similar to Ras, Myc overexpression alone is not sufficient for tumorigenesis since sustained Myc signaling activates cellular checkpoint regulators [128]. 

Myc is known to regulate a variety of transcription factors that subsequently promote mitochondrial biogenesis. For example, Myc induces mitochondrial biogenesis in murine hearts in part by inducing the expression of mitochondrial transcription factor A (TFAM) and peroxisome proliferator-activated receptor γ (PPARγ) coactivator-1α (PGC-1α) [134,135]. The role Myc plays in mitochondrial biogenesis has sparked interest into a potential role in mitochondrial dynamics. In Myc^−/−^ MEFs, the re-expression of Myc increases protein levels of Mfn2, Opa1, and Drp1 while promoting mitochondrial fusion [136]. These findings suggest that although expression of both the fusion and fission machinery is induced by Myc, it also regulates the activity levels of the machinery to tip the balance towards fusion. Consistent with this, the overexpression of Myc family member N-Myc leads to increased mitochondrial fusion in neuroblastoma cells [137]. Forcing fission in the context of inducible c-Myc expression results in decreased ATP production, suggesting that Myc-regulated fusion helps maintain proper cellular bioenergetics and ATP levels [138]. Additional studies in lymphoma cells have demonstrated that c-Myc promotes the utilization of glutamine in the tricarboxylic acid (TCA) cycle via increased expression of mitochondrial glutaminase for ATP production [139].

A recent study done in normal mammary and breast cancer cells showed that Myc promotes mitochondrial fusion by promoting phospholipase D family member 6 (PLD6) activity and that this fusion facilitates mitochondrial bioenergetics [140]. They further showed that Myc-driven mitochondrial fusion activates AMPK. This AMPK activation resulted in Yes-associated protein (YAP)/transcriptional coactivator with PDZ-binding motif (TAZ) inhibition, which is proposed to maintain the balance between MYC-driven cellular growth and YAP/TAZ-driven clonogenic growth, a characteristic of stem-like cells. AMPK activation also potentially acts as a rheostat to promote re-fragmentation of the fused mitochondrial network to help maintain a healthy mitochondrial population. Consistent with this potential role for mitochondrial dynamics in the maintenance of stemness, it has been demonstrated that Drp1-mediated mitochondrial fission is needed for growth, self-renewal, and tumor-forming ability for brain tumor initiating cells, a population of cells that exhibits stem cell-like properties [82]. In combination, these studies, using distinct model systems, demonstrate that mitochondrial fusion can inhibit stemness while mitochondrial fission promotes a dedifferentiated, stem-like state [70,82] and together suggest that mitochondrial fragmentation promotes cellular changes that support a more stem-like phenotype, regardless of the source of oncogenic signaling. 

It is intriguing that Ras and Myc, two strong drivers of oncogenic growth, appear to promote divergent mitochondrial morphologies. This is likely a consequence of the different sets of signaling pathways downstream of each oncogene [141]. Myc controls the expression of a large set of genes that promote cellular proliferation, a bioenergetically expensive process [127]. Since elongated mitochondria are associated with more efficient oxidative phosphorylation and the generation of ATP as well as macromolecules, it makes sense that a pro-proliferative oncogene such as Myc would promote mitochondrial elongation to take advantage of that efficiency [58,107]. It is less intuitive why Ras, which also promotes proliferation, would promote a less bioenergetically efficient morphology. Fragmented mitochondrial morphology is not limited to Ras-driven tumors like melanoma and pancreatic cancer, as mitochondrial fragmentation is seen in other tumor types like gliomas, hepatocellular carcinomas, and breast cancers [51,53,61,62,67,74,142]. It is clear from these studies demonstrating an increase in mitochondrial fragmentation in patient tumors of various tumor types that the physiological advantages of fission, some of which are explored above, must be sufficient to overcome this loss of efficiency provided by a more reticular mitochondrial network. 

## 8. Hypoxic Signaling

Many cancer cells in solid tumors experience hypoxic conditions. Hypoxia stabilizes inducible factor 1α (HIF-1α), a transcription factor that drives the expression of many genes, including those involved in the angiogenesis and metabolic reprogramming observed in cancer [143]. The relationship between mitochondrial dynamics and oxygen sensing is unsurprising given that both Drp1 and Mfn2 null mice lack trophoblast giant cell layers, which need adequate oxygen supplies for proper development [144]. In neuronal systems, hypoxia is known to induce mitochondrial fission [145,146]. In pulmonary artery smooth muscle cells, hypoxia has been shown to induce mitochondrial fission downstream of HIF-1α, [147]. That study also showed that activated HIF-1α mediates mitochondrial fission by CDK1-dependent phosphorylation of Drp1 [147]. Consistent with these findings, hypoxia induces mitochondrial fission and increased expression of Drp1 in glioblastoma cell lines [124]. Han et al. showed that hypoxia also induced Drp1 expression and mitochondrial fission in breast cancer cell lines [148].

Like oncogene-induced fission, hypoxia-induced fission can promote physiological changes that drive tumor progression. For example, hypoxia-induced fission was shown to promote cell migration in cancer cell lines [124,148]. It is also possible that hypoxia induced fission facilitates mitophagy to promote cellular survival, as was demonstrated in a neuronal system [149]. Marsboom et al. demonstrated that inhibition of HIF-1α or Drp1 reduces proliferation in pulmonary artery smooth muscle cells, suggesting that mitochondrial fragmentation can promote proliferation [147]. Furthermore, hypoxia induced fission may contribute to angiogenesis, as Drp1 was shown to mediate hypoxia-induced increases in pulmonary microvessels, and knockdown of Drp1 decreases CD31-positive blood vessels in a Ras-driven xenograft model [61,150]. In addition to these hypoxia-induced changes, HIF-1α has been shown to cooperate with other signaling nodes to promote pro-tumorigenic physiological changes. For example, HIF-1α was shown to cooperate with c-Myc in a lymphoma model to promote a Warburg phenotype in part by inducing the expression of pyruvate dehydrogenase kinase, a negative regulator of pyruvate dehydrogenase whose activity reduces pyruvate entry into the mitochondria and ultimately mitochondrial respiration [151]. Taken together, these data further support a pro-oncogenic role for hypoxia-induced mitochondrial fission in tumors. 

## 9. Conclusions

**Figure 3:**
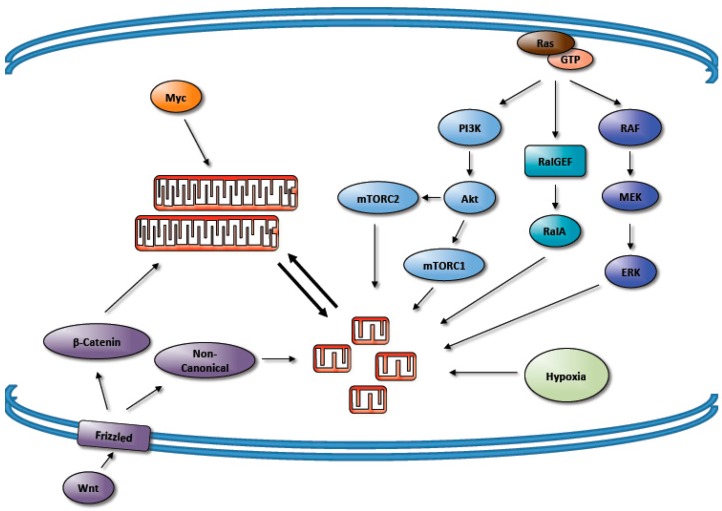
Oncogenic signals regulate the mitochondrial dynamics machinery to drive changes in mitochondrial morphology. Oncogenic MAPK, PI3K/Akt, and Ras-like guanine nucleotide exchange factor (RalGEF) signals promote mitochondrial fragmentation downstream of oncogenic Ras. Myc activity and canonical Wnt signaling, on the other hand, promote mitochondrial fusion, while non-canonical Wnt signaling and hypoxia induce fragmentation.

It is becoming increasingly apparent that many of the most commonly mutated oncogenic signaling networks converge upon the mitochondrial machinery to promote changes in mitochondrial morphology. Furthermore, it is evident that these changes in mitochondrial morphology play an important role in the tumorigenic process. Three distinct effector pathways downstream of oncogenic Ras, MAPK, PI3K/Akt, and RalGEF, have each been shown to directly activate Drp1 and promote mitochondrial fission. In addition, hypoxic conditions induce mitochondrial fission. Consistent with this, a variety of tumor specimens exhibit fragmented mitochondria or expression patterns consistent with it. Together, this demonstrates that numerous signals that are commonly seen in cancer promote fragmented mitochondrial morphology (Figure 3).

The observation of increased mitochondrial fission in tumors however is not universal and is likely to be dependent on cellular context. For example, Myc signaling promotes mitochondrial elongation as does the activation of canonical Wnt signaling, though non-canonical Wnt signaling was shown to be associated with the activation of Drp1 and mitochondrial fission [152]. Additionally, the inactivation of PTEN ablated the mitochondrial fusion induced by WNT/β-catenin signaling, showing how tumor suppressors may also modulate mitochondrial morphology. It will be important to explore the physiological consequences of these different strategies, especially given the amount of cross-talk between these pathways.

The fragmented morphology observed in the majority of tumor types appears to promote tumorigenesis through a variety of different routes. Fragmented mitochondria are permissive for appropriate cellular proliferation, promote a glycolytic metabolic phenotype, and promote cellular migration. The extensive mitochondrial fragmentation may also help cancer cells escape apoptosis, despite mitochondrial fission closely following apoptosis. Additionally, mitochondrial fission appears to promote a stem cell like phenotype, suggesting a potential role for mitochondrial fission in cancer stem-like cells. Although a growing amount of studies have begun to explore the role of mitochondrial fragmentation in tumorigenesis, the complexity of mitochondrial biology and the different model systems and approaches used have left the specific pro-tumorigenic mechanisms of mitochondrial fission unclear. Understanding the physiologic functions of mitochondrial fission is of critical importance to determine if manipulation of the mitochondrial morphology can be exploited as a viable therapeutic option.

To that end, additional factors will have to be considered if mitochondrial dynamics is to be a therapeutic target. For instance, there are likely to be effects of previous chemotherapeutics or targeted therapies on mitochondrial dynamics. There is some evidence showing that resistance to platinum-based therapy may arise due to mitochondrial fusion, while an enhancer of cisplatin activity may mediate its effects through increased mitochondrial fission [153,154]. Additionally, small molecule inhibitors of mitochondrial dynamics will also have effects on the tumor microenvironment. A study in cancer-associated myofibroblasts found that mitochondrial fission via overexpression of mitochondrial fission factor, an adaptor that facilitates fission, promotes glycolytic reprogramming and enhanced tumor growth [155]. Additionally, there is a growing body of literature looking at the role of mitochondrial dynamics in immune cells, another component of the tumor microenvironment [156]. Despite these advances in knowledge, many of these studies were performed outside of a physiologically relevant cancer context. Thus, greater research investigating mitochondrial fusion and fission is needed to understand the role of mitochondrial dynamics in cancer as well as general cell biology.
